# Editorial: Staving off gestational diabetes: Pancreatic islet adaptations and the extrinsic signals that drive them

**DOI:** 10.3389/fendo.2022.1056352

**Published:** 2022-11-23

**Authors:** James E. Bowe, Carol Huang, Henry Dong, Ronadip R. Banerjee

**Affiliations:** ^1^ Department of Diabetes, School of Cardiovascular and Metabolic Medicine and Sciences, King's College London, London, United Kingdom; ^2^ Department of Pediatrics, Cumming School of Medicine, University of Calgary, Calgary, AB, Canada; ^3^ Department of Pediatrics, University of Pittsburgh, Pittsburgh, PA, United States; ^4^ JDepartment of Medicine, Div of Endocrinology, Johns Hopkins University School of Medicine, Baltimore, MD, United States

**Keywords:** pancreatic islet, beta-cell, pregnancy, gestational diabetes, glucose metabolism, 11 intra-organ crosstalk, endocrinology 12

Preg nancy presents the maternal metabolism with the challenge of providing energy to the growing fetus whilst maintaining fuel homeostasis in the mother. A progressive increase in maternal insulin resistance over the course of gestation must be countered by increased insulin secretion to maintain normoglycemia. This increased insulin secretory capacity is met through functional changes in islet β-cells, including enhanced glucose-induced insulin secretion and increased β-cell mass. A failure of the β-cells to sufficiently compensate for the metabolic demand in pregnancy may lead to maternal glucose intolerance, hyperglycaemia, and Gestational Diabetes Mellitus (GDM). The prevalence of GDM in pregnant has increased in parallel with Type 2 Diabetes, and between 13-25% of pregnancies are currently estimated to be affected by GDM (1–4). GDM represents a threat to both mother and child and is associated with complications such as high blood pressure, preeclampsia, preterm birth, and macrosomia. Furthermore, approximately half of women with GDM subsequently develop Type 2 Diabetes. Children of GDM pregnancies are at increased risk of obesity and developing Type 2 Diabetes in later life. Thus, the major healthcare concerns for individuals diagnosed with GDM and their offspring drive the clinical need to identify novel strategies for diagnosing and treating the condition more effectively. To do so, we feel it is critical to first better understand the cellular mechanisms involved in the β-cell adaptation to pregnancy and the maternal signals that drive the adaptive response, which motivated the development of this Research Topic.

In response to our call for papers, we highlight 5 original research publications and two review articles accepted for publication. These encompass a diverse array of topics from calcium flux (Pretorius and Huang) and metabolomics of gestational islets and plasma (Zhang et al.), to the discovery of a role for the growth factor pleiotrophin in the gestational expansion of islets (Sevilliano et al.) and expanded mechanistic understanding of how prolactin (Aung et al.) and fatty acids (Kim et al.) act as extrinsic signals communicating pregnancy status to the islets. Furthermore, the description of a circular RNA (Zhang et al.) dysregulation in women with GDM, along with a comprehensive review by Zhang et al. on non-coding RNAs, and extracellular vesicles illustrate the potential for these circulating molecules to serve as biomarkers or therapeutic targets for GDM.

Original research from Zhang et al. evaluates the whole-body and islet-specific metabolic changes that occur during pregnancy. The authors observe enriched islet amino acid metabolism throughout pregnancy and upregulated glycerophospholipid and fatty acid metabolism during late-pregnancy. Evidence linking specific amino acids to enhanced insulin secretion, and data linking specific amino acids and lipids to effects on β-cell proliferation, led the authors to propose that altered islet metabolism may mediate the adaptive changes in insulin secretion and potentially β-cell mass. Sevilliano et al. identify pleiotrophin as a novel local mechanism that is potentially involved in β-cell adaptations to pregnancy. Pleiotrophin, a heparin-binding cytokine, is expressed by both human and murine islets and induces increased DNA synthesis and Pdx1 expression in the INS1E cell line, results that the authors posit are consistent with a potential role of pleiotrophin in β-cell expansion.

Several different classes of signaling molecules, including hormones, metabolites, and non-coding RNAs, have been implicated in communication from other tissues to the islets during pregnancy. It is well established that prolactin and placental lactogen play a critical role in driving the β-cell adaptive response *via* the prolactin receptor. Several recent studies have confirmed the critical role of the β-cell prolactin receptor (Prlr) in gestational adaptation 1–3. However, because of the technical aspects of the development of these genetic knockout models, some PRLR knockdown in the hypothalamus might have contributed to the observed phenotypes *via* central actions of prolactin. Aung et al. address this possibility, and through the use of forebrain-specific Prlr knockout mice demonstrate conclusively that the previously reported effects of prolactin on islet adaptations are not indirect central effects. This reinforces the importance of prolactin and placental lactogen acting directly on the β-cells in driving the adaptive response to pregnancy. Other non-hormonal circulating factors also influence β-cell adaptation; Kim et al. present novel data examining the role of fatty acid (FA) signaling in gestational adaptation. They show augmented insulin secretion in the presence of fatty acids and altered metabolic partitioning within the β-cells, with increased FA esterification, and reduced FA oxidation, hence concluding that the elevated circulating fatty acids present during pregnancy also play a role in driving islet adaptations. In another review, Pretorius and Huang provide an overview of recent data suggesting that altered intracellular calcium dynamics during pregnancy may mediate enhanced insulin secretion and β-cell proliferation. They propose that dysfunctional intracellular calcium dynamics may contribute to GDM development and provide a compelling case for further research in this field.

In addition to classical signal transduction driven by peptide hormones, lipids, and intracellular calcium, recent research has highlighted potential roles for non-coding RNAs and extracellular vesicles in the pathogenesis of GDM, here reviewed by Zhang et al. Whilst the pathophysiological mechanisms involved are largely unknown, an increasing number of long non-coding RNAs, microRNAs, and circular RNAs appear to be differentially expressed in GDM. The review also covers the role of extracellular vesicles as carriers mediating communication between tissues, indicating that altered vesicle cargo may play a role in GDM development. New research from another group identifies circ_0001578, a non-coding circular RNA present in the placenta and in circulating extracellular vesicles, as a specific predictor of GDM development (Zheng et al.). Together, these studies present a compelling case for further research into the potential use of specific non-coding RNAs and extracellular vesicle analysis as early biomarkers for GDM development.

These papers advance our understanding of gestational adaptation within islets and aid in the identification of biomarkers of GDM, but the challenge of translating molecular findings in murine models to human biology remains. Conversely, human studies show the role of the dysregulation of circulating markers in GDM, but the mechanistic explanations for a normal physiologic role for these molecules are still unclear. Cross-pollination and collaboration between groups identifying novel targets in murine models, and those discovering the markers observed in human GDM are critical if we are to achieve the goal of expanding molecular understanding of gestational adaptations sufficiently to promote rational changes in the diagnosis and therapy of human GDM.

## Author contributions

All authors listed made a substantial, direct contribution to the work and approved its publication.

## Conflict of interest

The authors declare that the research was conducted in the absence of any commercial or financial relationships that could be construed as a potential conflict of interest.

## Publisher’s note

All claims expressed in this article are solely those of the authors and do not necessarily represent those of their affiliated organizations, or those of the publisher, the editors and the reviewers. Any product that may be evaluated in this article, or claim that may be made by its manufacturer, is not guaranteed or endorsed by the publisher.
